# Role of Sleep Duration and Timing on Paediatric BMI Across Childhood and Adolescence: Do Both Matter?

**DOI:** 10.1111/ijpo.70064

**Published:** 2025-10-21

**Authors:** Yundan Zhang, Joyce M. Lee, Karen E. Peterson, Jonathan A. Mitchell, Erica C. Jansen

**Affiliations:** ^1^ Department of Applied Health Science School of Public Health, Indiana University Bloomington Indiana USA; ^2^ Division of Pediatric Endocrinology, Susan B. Meister Child Health Evaluation and Research Center University of Michigan Ann Arbor Michigan USA; ^3^ Department of Nutritional Sciences School of Public Health, University of Michigan Ann Arbor Michigan USA; ^4^ Department of Environmental Health Sciences School of Public Health, University of Michigan Ann Arbor Michigan USA; ^5^ Division of Gastroenterology, Hepatology and Nutrition Children's Hospital of Philadelphia Philadelphia Pennsylvania USA; ^6^ Department of Pediatrics University of Pennsylvania Philadelphia Pennsylvania USA

**Keywords:** circadian, electronic health record, longitudinal, paediatric, sleep duration

## Abstract

**Background:**

Adequate sleep duration is a prevention factor for paediatric obesity, but the role of timing is still unclear.

**Objectives:**

To investigate associations of sleep duration and timing with BMI in a large paediatric database.

**Methods:**

Medical chart and survey data were collected from 29 409 children aged 2–18 years who attended well‐child visits between Jan 2019 and Dec 2023 (repeated‐measures cross‐sectional design). Logistic and linear mixed effects regression models accounting for repeated measures estimated odds of overweight/obesity and continuous BMI‐for‐age CDC‐based percentiles for each additional/later hour of sleep duration, midpoint (median of bedtime and wake time), and bedtime, adjusted for potential confounders and stratified by age groups.

**Results:**

Among young children (2–5 years), shorter sleep duration but not sleep timing was related to higher odds of overweight/obesity (21% higher odds with 95% CI: 3% to 36%). In mid‐childhood (6–12 years), shorter sleep duration and later midpoint were associated with higher odds of overweight/obesity (18%, 95% CI = 9%, 26%; 32%, 95% CI = 17%, 49%). Among adolescents (13–18 years), each hour of later sleep midpoint equated to 12% higher odds of living with overweight/obesity (95% CI: 1% to 23%). Linear models were similar.

**Conclusions:**

Shorter sleep duration at younger ages and later sleep timing in adolescence were associated with higher BMI.

## 1 Introduction

Adequate sleep duration is now recognised as an important component of healthy weight status in paediatric populations [1]. Yet, sleep duration is only one facet of overall sleep health, and the roles of other related aspects of sleep in body weight regulation are less clear. In particular, sleep timing, for example, bedtimes, wake times, or midpoint of sleep, could be relevant due to its role in modulating circadian rhythms. Specifically, metabolism and energy balance may be disrupted when the timing of sleep is out of sync with internal circadian rhythms, often described as circadian misalignment [2, 3]. Nonetheless, sleep timing has shown inconsistent associations with weight status among youth. Whereas one review of 20 observational paediatric studies reported weak evidence for an independent effect of later sleep timing on adiposity [4], a subsequent review focused on bedtime reported a 27% higher pooled odds for the association between later bedtime and obesity among 12 studies [5]. Moreover, the latter study reported similar associations with and without adjustment for sleep duration, suggesting a potential sleep timing effect independent of sleep duration.

One possible explanation for apparent discrepancies in the literature is that sleep duration and timing relationships with BMI are dependent on age and stage of development [6]. Sleep duration and timing change considerably across childhood. For example, from early childhood to mid‐childhood, there are transitions out of daily napping to longer and more consolidated nighttime sleep [7]. From mid‐childhood to adolescence, there are declines in usual sleep duration and delays in sleep timing that coincide with pubertal onset and progression [8]. Furthermore, there is a strong interplay between sleep duration and timing that depends on family and school schedules, which also varies considerably in younger versus older children. Among US high school students, late bedtimes and short sleep duration are typically highly positively correlated, at least during school nights, since sleep time is truncated by early school start times [9]. Given the changing and interrelated nature of sleep duration and timing relationships across childhood and adolescence, there is a need to consider the independent relationships of sleep duration and timing with adiposity stratified by age categories.

Moreover, there are direct clinical applications to uncovering the independent roles of sleep duration and timing on adiposity in childhood. Specifically, is it relevant to ask about and potentially intervene on both sleep duration and sleep timing, or is it sufficient to focus solely on sleep duration? In the 2023 Clinical Practice for Evaluation and Treatment for Children and Adolescents with Obesity from the American Academy of Paediatrics, achieving adequate sleep duration is the primary focus when evaluating and treating children living with obesity [1]. If sleep timing were to demonstrate an additional measurable impact on obesity risk, this would suggest the need for revised guidelines and interventions that encompass multiple facets of sleep health for prevention and treatment of obesity among children of different ages. To address these research questions, we utilised a large US‐based EHR dataset of > 25 000 paediatric patient records that included self‐ and caregiver‐reported sleep information. With this large dataset of repeated measures, we aimed to determine if sleep duration and timing were independently associated with paediatric obesity across multiple developmental age periods within the US context.

## 2 Methods

### 2.1 Study Population and Design

**FIGURE 1:**
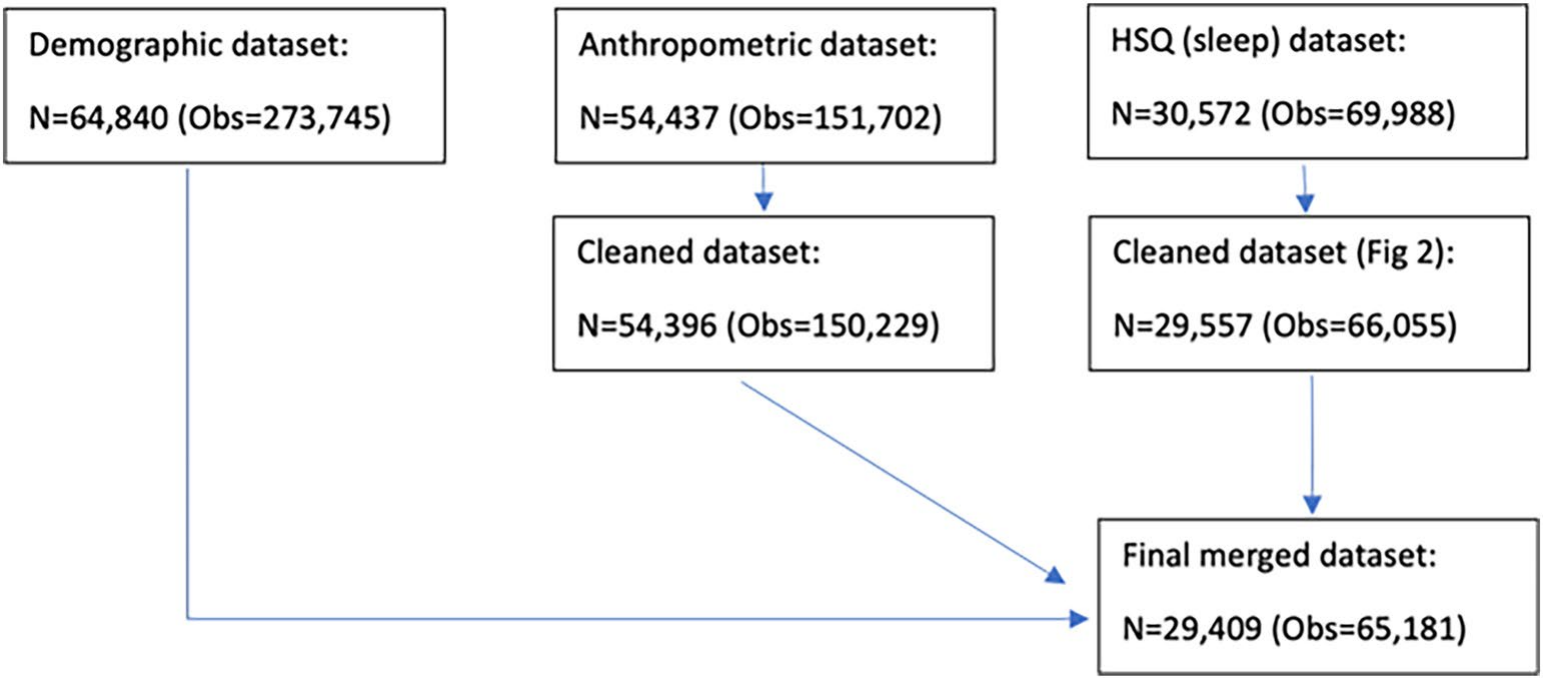
Curation of final dataset.

Electronic Health Record (EHR) data were extracted for paediatric patients aged 0 to 17 years old seen in primary care who had a Well Child/Health maintenance exam between January 2019 and December 2023 at an academic medical system located in Southeast Michigan. Well child visits are regular check‐ups that allow healthcare providers to address concerns raised by parents and patients, evaluate the child's environment, identify developmental and health issues, and follow up on ongoing conditions through a comprehensive approach including screening tests, growth evaluation, and personalised care plans. At the yearly visits, patients and/or their caregivers complete a health and safety questionnaire (HSQ) which included sleep questions to serve as a screener for providing adequate anticipatory guidance. Within the EHR database, 64 840 patients had demographic and encounter data between 01/01/2019 and 12/31/2023. Among these, 54 543 patients had anthropometric data and 30 572 patients had completed the sleep items within the HSQ. Due to very different sleep needs and patterns in the first few years of life [10], we only included patients aged older than 2 years old. After excluding or correcting implausible values and merging the three data sources (demographic, anthropometric, and HSQ), the final cohort of this study included 29 409 patients aged 2 to 18 years old with 65 181 observations (at least 1 visit and up to 6; Figure 1). The study design is characterised as a repeated‐measures cross‐sectional design, given that each patient could have contributed up to 6 data points. Detailed data collection and cleaning processes of the three datasets are explained below. The Institutional Review Board at the University of Michigan approved the study (HUM00244211).

### 2.2 Anthropometric Measurements

Weight and height are measured at the clinical visit by medical assistants as part of standard well‐child care. To clean the height and weight data, we used the R package growthcleanr described by Daymont [11, 12, 13], which follows an automatic rule‐based cleaning process designed specifically for longitudinal weight and height data that considers both individual‐level growth and population‐level criteria, based on CDC reference growth curves [14]. The process relies on the comparison between the weight and height standard deviation (SD) scores and the weighted moving average to identify implausible weight and height values over time, using an inverse‐distance weighting. Other types of errors such as unit error, carry‐forward value, duplicates, height absolute differences, and single or paired measurement error are also considered. Within the original anthropometric dataset (i.e., before merging with demographic and sleep data), a total of 1473 exclusions were made (55% of the excluded data were carry‐forward values). Detailed drop‐out numbers and the categories of exclusion can be found in the Table S1.

Body mass index (BMI) was calculated as weight (kg) divided by height (m^2^). BMI percentiles accounting for age and sex were calculated using the cdcanthro R package developed by the Center for Disease Control and Prevention (CDC) (https://github.com/CDC‐DNPAO/CDCAnthro). BMI percentiles were calculated using the LMS method based on the CDC 2000 growth charts [14]. We opted to use the CDC growth reference given that this is a US population and to aid in the interpretation of these clinical data. Overweight and obesity definitions were classified based on CDC guidelines; overweight was defined as a BMI from the 85th to less than the 95th percentile, and obesity was defined as a BMI at or above the 95th percentile [14, 15]. We did not consider underweight and normal weight as separate groups in our analysis due to small numbers in the underweight category (see Table S3 for proportions of weight categories according to age).

### 2.3 Sleep Health Assessment

Usual weekday bedtime and wake time were recorded in HSQ in a 24‐h format in response to the prompt: “On an average weekday, what time does your child/do you: Go to sleep? Wake up?”. These questions were adapted from the validated Children's Sleep Habits Questionnaire [16].

These decimal time variables were reported by the parent for paediatric patients 10 years or younger and by the paediatric patient if they were 11 years or older. We developed two numerical variables related to sleep: sleep duration and sleep midpoint, both derived from bedtime and wake time. Sleep duration was calculated as the total number of hours between bedtime and wake time. Sleep midpoint, which is a marker of sleep timing and a proxy for circadian misalignment, was calculated as the median clock time between bedtime and wake time. For example, a bedtime of 12 AM and wake time of 8 AM would yield a midpoint of 4 AM. We also examined bedtime as a separate exposure, given that bedtime is not constrained by school and work start times and therefore may be more amenable to change than wake times.

The cleaning process for the sleep variables is shown in Figure S1. Out of 30 572 patients with at least one report of bedtime and waketime (*n* = 69 988 total observations), 2871 observations with missing values either in bedtime or wake time, and 289 observations with identical time in bed and wake were excluded. The remaining 66 868 observations were categorised as 1: plausible nocturnal sleep pattern (*n* = 63 058), 2: probable nap (*n* = 10), 3: night shift (*n* = 47), 4: entry error (*n* = 3528), and 5: other types of error (*n* = 225). Figure 1 shows the details for each of the criteria. Observations meeting the criteria for categories 2 (nap), 3 (night shift), and 5 (other types of error) were excluded. Observations meeting the criteria for 4 (entry error) were corrected as shown in Figure 1 (e.g., AM changed to PM). Finally, we excluded 541 observations that were aged under 2 years old. The final sleep dataset included 29 557 individuals with 66 055 observations.

In addition to treating the sleep duration as a continuous variable, we created a categorical variable, “Meet Sleep Recommendation” to assess whether individuals met the recommended sleep duration according to age group guidelines set by the American Academy of Sleep Medicine [10]. The criteria are as follows: for children aged 2–5, the recommended sleep duration is 10–13 h per day; for ages 6–12, it is 9–12 h per day; and for adolescents aged 13–18, the recommended duration is 8–10 h. This variable is coded as 0 if the recommended sleep duration is not met and 1 if it is met or exceeded. Those who exceeded duration recommendations were not classified as a separate group; they constituted less than 5% of the total population.

### 2.4 Covariates

Potential confounders were selected a priori for plausible associations with both sleep and BMI. These included the following demographic characteristics: age, sex (Male, Female), and race (white or Caucasian, Black or African American, Asian, Other). Meteorological season (Spring, Summer, Autumn, Winter) at encounter was created using the encounter date to adjust for possible seasonal influence on sleep and BMI. Specifically, winter was defined as the period from December 1st through February 29th, spring from March 1st through May 31st, summer from June 1st through August 31st, and autumn from September 1st through November 30th [17]. Lifestyle factors, including dietary intake and screen time, were assessed using the HSQ. Diet quality was assessed through usual intake of four foods considered as proxies of dietary quality: fruit intake (≤ 1 serving/day, 2 servings/day, ≥ 3 servings/day), vegetable intake (≤ 1 serving/day, 2–3 servings/day, ≥ 4 servings/day), sugar‐sweetened beverage intake (0 servings/day, 1 serving/day, 2 servings/day, or ≥ 3 servings/day), and fast food intake (0 servings/day, 1–2 servings/day, ≥ 3 servings/day). Screen time was defined as the total daily hours a child spends on electronic devices, categorised as 0–1 h/day, 1–2 h/day, and > 2 h/day. For instances where lifestyle factor data were missing in certain encounters, values from previous assessments were carried forward if available.

### 2.5 Statistical Analysis

The analytic sample was stratified into three age groups corresponding to different sleep requirements across childhood: Group 1 includes children aged 2–5 years, Group 2 comprises children aged 6–12 years, and Group 3 consists of adolescents aged 13–18 years. We first summarised baseline demographic characteristics using means and standard deviations (SD) for continuous variables, and proportions for categorical variables. To determine differences in baseline sleep characteristics between categories, we utilised ANOVA tests and visualised the data by plotting the distribution of sleep duration and sleep midpoint across the three age groups.

Linear mixed‐effects (LME) models were utilized to evaluate associations between sleep duration and sleep midpoint on BMI percentile. Logistic regression was employed to examine the association between sleep patterns and categorical weight outcomes, categorised as underweight/normal and overweight/obesity. Consideration of more than two weight categories (i.e., ordinal logistic model) was not possible due to issues with model convergence. Both linear and logistic analyses incorporated a base model that adjusted for age, sex, season, and race. Fully adjusted models included fruit and vegetable intake, sugar‐sweetened beverage consumption, and screen time. Both sets of analyses were stratified by age group. For Age Groups 1 and 2, sleep duration and sleep midpoint were included in the same models. For Age Group 3, sleep duration and sleep midpoint were adjusted independently because of high correlation between these two variables. In sensitivity analyses, we explored possible U‐shaped associations and stratification by sex. Interaction tests of sleep characteristics with age groups were also run to evaluate effect modification due to the pre‐specified age groups. Finally, we conducted sensitivity analysis within Group 2, splitting into ages 6–10 years vs. 11–12 years, to account for the strong likelihood that a proportion of children older than 10 years will have already initiated puberty.

## 3 Results

The study sample included 29 409 children aged 2–18 (50.6% male). The average age of the first encounter was 8.8 (4.4) years, and each patient contributed an average of 2 visits (range: 1, 6) to the dataset (Table 1). The data included 10 034 patients (34%) aged 2 to 5 years, 12 428 patients (42%) aged 6 to 12 years, and 6947 patients (24%) aged 13 to 18 years. The majority of patients reported they were White/Caucasian (79%). Prevalence of overweight/obesity varied by age (12% at age 2% and 28% at age 18) but on average was close to 30% (Figure S3). Based on self‐ or caregiver‐reported bedtimes and wake times, 11% of children ages 2–5, 9% of children 6–12, and 30% of children 13–18 did not meet sleep duration recommendations for their age at the time of their first well child visit.

**TABLE 1:** Characteristics of the paediatric primary care population.

	Overall sample (obs = 65 181)	Preschool (age 2–5) (obs = 13 753)	Children (age 6–12) (obs = 34 350)	Adolescents (age 13–18) (obs = 17 068)
*N*	29 409	10 034	17 004	9769
Age (First Encounter), years, mean (SD)	8.76 (4.35)	4.29 (0.57)	8.10 (2.09)	14.63 (1.62)
Sex (Male), *N* (%)	14 894 (50.64)	5198 (51.80)	8757 (51.38)	4778 (48.91)
Race/ethnicity, *N* (%)				
White or Caucasian	23 226 (78.98)	7933 (79.06)	13 385 (78.53)	7951 (81.39)
Black or African American	2484 (8.45)	830 (8.27)	1421 (8.34)	749 (7.67)
Asian	1806 (6.14)	634 (6.32)	1019 (5.98)	585 (5.99)
Other	1893 (6.44)	637 (6.35)	1219 (7.15)	484 (4.95)
State of residence (MI), *N* (%)	29 042 (98.82)	9910 (98.85)	16 855 (98.97)	9679 (99.11)
Number of visits, mean (min, max)	2.22 (1, 6)	1.37 (1, 4)	2.02 (1, 5)	1.75 (1, 5)

**FIGURE 2:**
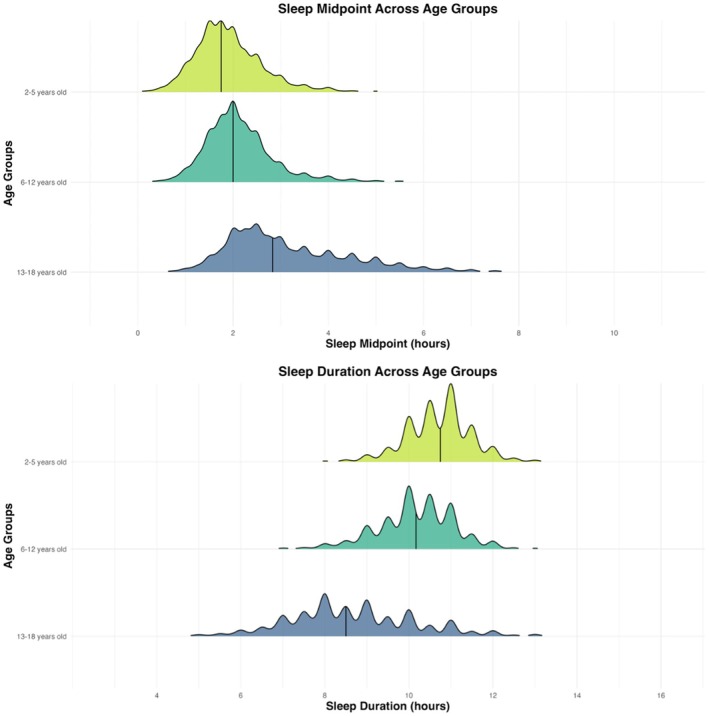
Sleep midpoint and duration by age groups in a paediatric primary care population.

Average sleep duration was inversely associated with age, and average sleep timing was positively associated with age, as shown in Figure 2 (sleep duration and midpoint) and Figure S2 (bedtime and wake times, and visualisations of correlations between duration and midpoint). Shorter sleep duration and later sleep timing were associated with non‐White race/ethnicity, lower fruit and vegetable intake, higher sugar‐sweetened beverage and fast‐food intake, and higher screen time (Table 2). Associations between sleep duration and sex depended on age group, with 2–5‐year‐old males having a 5‐min shorter sleep duration, but adolescent males (13–18 years) having a 4‐min longer sleep duration than females (no difference in middle childhood). For sleep timing, males had earlier sleep times (approximately 4 min) than females across age categories.

**TABLE 2:** Sleep duration and timing according to sociodemographic and lifestyle characteristics in a paediatric primary care population^a^.

	Sleep duration (mean ± SD)	Bedtime (mean ± SD)	Wake time (mean ± SD)	Midpoint (mean ± SD)
Preschool				
Ages 2–5 (*N* = 10 034)				
Sex				
Female	10:45 ± 00:48	20:35 ± 00:50	07:20 ± 00:52	01:57 ± 00:45
Male	10:40 ± 00:48***	20:34 ± 00:49	07:14 ± 00:52***	01:53 ± 00:44***
Race				
White	10:46 ± 00:46	20:29 ± 00:46	07:14 ± 00:49	01:51 ± 00:42
Black	10:28 ± 01:05	21:02 ± 00:58	07:29 ± 01:09	02:16 ± 00:55
Asian	10:26 ± 00:48	21:02 ± 00:55	07:29 ± 00:52	02:16 ± 00:47
Other	10:40 ± 00:50***	20:46 ± 00:54***	07:25 ± 00:56***	02:05 ± 00:49***
Fruit intake				
≤ 1/d	10:34 ± 00:56	20:52 ± 00:56	07:25 ± 01:00	02:08 ± 00:51
2/d	10:41 ± 00:48	20:37 ± 00:49	07:18 ± 00:52	01:58 ± 00:44
≥ 3/d	10:46 ± 00:46***	20:29 ± 00:47***	07:14 ± 00:50***	01:52 ± 00:43***
Vegetables intake				
≤ 1/d	10:38 ± 00:49	20:40 ± 00:52	07:18 ± 00:53	01:59 ± 00:47
2–3/d	10:44 ± 00:47	20:32 ± 00:48	07:16 ± 00:51	01:54 ± 00:43
≥ 4/d	10:46 ± 00:51***	20:29 ± 00:49***	07:15 ± 00:55	01:52 ± 00:45***
Sweet beverages intake				
0/d	10:46 ± 00:44	20:25 ± 00:46	07:11 ± 00:47	01:48 ± 00:41
1/d	10:41 ± 00:49	20:38 ± 00:49	07:19 ± 00:52	01:58 ± 00:44
2/d	10:37 ± 00:53	20:54 ± 00:53	07:31 ± 01:02	02:13 ± 00:52
≥ 3/d	10:29 ± 00:56***	21:10 ± 01:00***	07:40 ± 01:11***	02:25 ± 00:56***
Fast foods intake				
0/d	10:45 ± 00:47	20:28 ± 00:49	07:13 ± 00:50	01:50 ± 00:43
1–2/d	10:43 ± 00:47	20:34 ± 00:48	07:17 ± 00:51	01:55 ± 00:44
≥ 3/d	10:32 ± 00:58***	21:02 ± 01:01***	07:34 ± 01:07***	02:17 ± 00:58***
Screen time				
0–1 h	10:47 ± 00:46	20:24 ± 00:45	07:11 ± 00:47	01:47 ± 00:40
1–2 h	10:40 ± 00:47	20:38 ± 00:47	07:19 ± 00:52	01:59 ± 00:44
> 2 h	10:31 ± 00:58***	21:05 ± 00:59***	07:35 ± 01:07***	02:20 ± 00:56***
Children				
Ages 6–12 (*N* = 17 044)				
Sex				
Female	10:10 ± 01:01	21:05 ± 00:55	07:16 ± 00:59	02:11 ± 00:48
Male	10:09 ± 00:55	21:04 ± 00:52***	07:13 ± 00:56***	02:08 ± 00:47***
Race				
White	10:11 ± 00:57	21:18 ± 00:52	07:13 ± 00:56	02:07 ± 00:46
Black	10:01 ± 01:08	21:17 ± 00:59	07:17 ± 01:10	02:17 ± 00:55
Asian	09:59 ± 00:56	21:25 ± 00:51	07:25 ± 00:51	02:25 ± 00:43
Other	10:11 ± 00:55***	21:08 ± 00:54***	07:19 ± 00:58***	02:13 ± 00:49***
Fruit intake				
≤ 1/d	09:56 ± 01:05	21:21 ± 00:59	07:17 ± 01:07	02:19 ± 00:54
2/d	10:09 ± 00:57	21:05 ± 00:52	07:14 ± 00:56	02:10 ± 00:46
≥ 3/d	10:17 ± 00:53***	20:55 ± 00:50***	07:13 ± 00:53***	02:04 ± 00:44***
Vegetables intake				
≤ 1/d	10:04 ± 01:01	21:13 ± 00:56	07:16 ± 01:02	02:14 ± 00:50
2–3/d	10:13 ± 00:55	21:00 ± 00:51	07:13 ± 00:54	02:07 ± 00:45
≥ 4/d	10:14 ± 00:58***	20:59 ± 00:54***	07:14 ± 00:56***	02:07 ± 00:47***
Sweet beverages intake				
0/d	10:16 ± 00:50	20:55 ± 00:50	07:10 ± 00:50	02:02 ± 00:43
1/d	10:08 ± 00:51	21:06 ± 00:51	07:15 ± 00:56	02:10 ± 00:46
2/d	10:00 ± 00:56	21:21 ± 00:56	07:21 ± 01:08	02:21 ± 00:53
≥ 3/d	09:46 ± 01:10***	21:40 ± 01:10***	07:26 ± 01:23***	02:33 ± 01:06***
Fast foods intake				
0/d	10:15 ± 00:56	20:58 ± 00:52	07:13 ± 00:55	02:06 ± 00:46
1–2/d	10:10 ± 00:57	21:04 ± 00:52	07:14 ± 00:56	02:09 ± 00:46
≥ 3/d	09:57 ± 01:10***	21:25 ± 01:02***	07:22 ± 01:11***	02:23 ± 00:57***
Screen time				
0–1 h	10:25 ± 00:48	20:41 ± 00:44	07:07 ± 00:44	01:54 ± 00:37
1–2 h	10:11 ± 00:53	21:02 ± 00:46	07:12 ± 00:52	02:07 ± 00:41
> 2 h	09:51 ± 01:09***	21:35 ± 01:00***	07:26 ± 01:14***	02:30 ± 00:58***
Adolescents				
Ages 13–18 (*N* = 9769)				
Sex				
Female	08:38 ± 01:36	22:53 ± 01:17	07:31 ± 01:48	03:12 ± 01:20
Male	08:41 ± 01:31*	22:50 ± 01:19**	07:31 ± 01:46	03:10 ± 01:22
Race				
White	08:41 ± 01:32	22:49 ± 01:16	07:29 ± 01:46	03:09 ± 01:20
Black	08:33 ± 01:47	23:06 ± 01:29	07:39 ± 01:58	03:22 ± 01:58
Asian	08:25 ± 01:25	23:08 ± 01:14	07:32 ± 01:37	03:20 ± 01:37
Other	08:40 ± 01:40***	22:57 ± 01:22***	07:37 ± 01:52**	03:17 ± 01:52***
Fruit intake				
≤ 1/d	08:33 ± 01:38	23:01 ± 01:24	07:34 ± 01:54	03:17 ± 01:28
2/d	08:40 ± 01:32	22:49 ± 01:14	07:29 ± 01:46	03:09 ± 01:19
≥ 3/d	08:46 ± 01:29***	22:43 ± 01:13***	07:29 ± 01:40**	03:06 ± 01:15***
Vegetables intake				
≤ 1/d	08:35 ± 01:38	23:01 ± 01:22	07:36 ± 01:55	03:18 ± 01:27
2–3/d	08:41 ± 01:29	22:45 ± 01:13	07:26 ± 01:40	03:05 ± 01:15
≥ 4/d	08:50 ± 01:34***	22:42 ± 01:15***	07:32 ± 01:45***	03:07 ± 01:18***
Sweet beverages intake				
0/d	08:41 ± 01:28	22:45 ± 01:16	07:26 ± 01:38	03:05 ± 01:16
1/d	08:40 ± 01:19	22:49 ± 01:15	07:29 ± 01:45	03:09 ± 01:19
2/d	08:37 ± 01:40	22:57 ± 01:20	07:34 ± 01:53	03:15 ± 01:24
≥ 3/d	08:35 ± 01:51*	23:13 ± 01:28***	07:47 ± 02:05***	03:30 ± 01:32***
Fast foods intake				
0/d	08:42 ± 01:28	22:44 ± 01:14	07:26 ± 01:40	03:05 ± 01:16
1–2/d	08:40 ± 01:32	22:49 ± 01:16	07:29 ± 01:46	03:08 ± 01:19
≥ 3/d	08:33 ± 01:45**	23:17 ± 01:27***	07:51 ± 02:02***	03:34 ± 01:32***
Screen time				
0–1 h	09:01 ± 01:23	21:57 ± 01:07	06:58 ± 01:22	02:28 ± 01:02
1–2 h	08:43 ± 01:25	22:29 ± 01:05	07:12 ± 01:31	02:51 ± 01:07
> 2 h	08:36 ± 01:37***	23:04 ± 01:19***	07:40 ± 01:53***	03:22 ± 01:25***

^a^ ANOVA tests used to determine statistical significance of difference in means across categories * indicates statistical significance at *p* < 0.05, ** indicates statistical significance at *p* < 0.01, *** indicates statistical significance at *p* < 0.001.

In logistic regression models, associations of sleep duration and timing with overweight/obesity (OWOB) depended on age group (P for interactions all < 0.001). Among young children, longer sleep duration but not sleep timing was related to lower odds of OWOB (Table 3), such that each additional hour of sleep was related to 21% lower odds of OWOB (95% CI: 3% to 36%). In mid‐childhood, longer sleep duration was associated with 18% lower likelihood of OWOB (95% CI: 9% to 26%), while later sleep midpoint and later bedtime were associated with 32% higher odds (95% CI: 17% to 49%) and 35% higher odds of OWOB (95% CI: 21% to 52%), respectively. Among adolescents, only sleep timing was related to OWOB, such that each hour later sleep midpoint equated to 12% higher odds of OWOB (95% CI: 1% to 23%), and each hour later bedtime resulted in 10% increased odds of OWOB (95% CI: 0% to 22%). Linear mixed models of sleep duration and timing in relation to continuous BMI percentiles were similar to the findings from the logistic models (Table 4). The only exception was that among adolescents aged 13–18, both sleep timing and sleep duration were associated with BMI percentile. Specifically, each additional hour of sleep duration was associated with a 0.28 lower BMI percentile (95% CI: −0.45 to −0.11; Table 4) and a 1‐h later bedtime was associated with a 0.29 higher BMI percentile (95% CI: 0.06 to 0.52).

**TABLE 3:** Logistic regression estimates between sleep duration and overweight/obesity in a paediatric primary care population.

	*N*	Odds of overweight/obesity, minimally‐adjusted estimate (95% CI)^a^	*p*	*N*	Odds of overweight/obesity, fully‐adjusted estimate (95% CI)^b^	*p*
Sleep duration, per hour longer						
Ages 2–5	10 034	0.76 (0.62, 0.92)	0.006	9936	0.79 (0.64, 0.97)	0.02
Ages 6–12	17 044	0.78 (0.71, 0.86)	< 0.001	16 916	0.82 (0.74, 0.91)	< 0.001
Ages 13–18^c^	9769	1.01 (0.93, 1.09)	0.86	9718	1.02 (0.94, 1.11)	0.63
Sleep midpoint, per hour later						
Ages 2–5	10 034	1.17 (0.93, 1.48)	0.19	9936	1.03 (0.81, 1.32)	0.79
Ages 6–12	17 044	1.43 (1.27, 1.62)	< 0.001	16 916	1.32 (1.17, 1.49)	< 0.001
Ages 13–18^c^	9769	1.14 (1.03, 1.25)	0.01	9718	1.12 (1.01, 1.23)	0.03
Bedtime, per hour later						
Ages 2–5	10 034	1.28 (1.05, 1.57)	0.02	9936	1.14 (0.92, 1.42)	0.24
Ages 6–12	17 044	1.46 (1.31, 1.65)	< 0.001	16 916	1.35 (1.21, 1.52)	< 0.001
Ages 13–18^c^	9769	1.14 (1.03, 1.26)	0.01	9718	1.10 (1.00, 1.22)	0.06

^a^ Includes adjustment for age, sex, season, race/ethnicity, and mutual adjustment for sleep duration and sleep midpoint.

^b^ Includes adjustment for age, sex, season, race/ethnicity, fruit/vegetable intake, sugar‐sweetened beverage intake, screentime, and mutual adjustment for sleep duration and sleep midpoint.

^c^ For oldest age group, sleep duration and midpoint were not included in the same models due to collinearity.

**TABLE 4:** Linear mixed effects regression estimates between sleep characteristics and BMI percentiles in a paediatric primary care population.

	BMI percentile, minimally‐adjusted estimate (95% CI)^a^	*p*	BMI percentile, fully‐adjusted estimate (95% CI)^b^	*p*
Sleep duration, per hour longer				
Ages 2–5	−0.61 (−1.11, −0.12)	0.02	−0.51 (−1.01, −0.01)	0.048
Ages 6–12	−0.85 (−1.07, −0.64)	< 0.001	−0.72 (−0.94, −0.50)	< 0.001
Ages 13–18^c^	−0.29 (−0.46, −0.12)	0.0009	−0.28 (−0.45, −0.11)	0.001
Sleep midpoint, per hour later				
Ages 2–5	0.34 (−0.27, 0.94)	0.27	−0.12 (−0.75, 0.50)	0.70
Ages 6–12	0.75 (0.47, 1.03)	< 0.001	0.45 (0.17, 0.73)	0.002
Ages 13–18^c^	0.10 (−0.12, 0.31)	0.37	0.03 (−0.19, 0.25)	0.77
Bedtime, per hour later				
Ages 2–5	0.55 (0.00, 1.10)	0.05	0.15 (−0.42, 0.72)	0.61
Ages 6–12	0.94 (0.68, 1.21)	< 0.001	0.65 (0.38, 0.92)	< 0.001
Ages 13–18^c^	0.37 (0.14, 0.59)	0.002	0.29 (0.06, 0.52)	0.01

^a^ Includes adjustment for age, sex, season, race/ethnicity, and mutual adjustment for sleep duration and sleep midpoint.

^b^ Includes adjustment for age, sex, season, race/ethnicity, fruit/vegetable intake, sugar‐sweetened beverage intake, screentime, and mutual adjustment for sleep duration and sleep midpoint.

^c^ For oldest age group, sleep duration and midpoint were not included in the same models due to collinearity.

In sensitivity analyses, we examined possible U‐shaped associations and sex differences, but neither U‐shaped associations nor interactions by sex were found. Moreover, for the first two age groups, we examined whether estimates appreciably changed when sleep duration and timing were analysed in individual models vs. the same model (i.e., mutual adjustment) and found that results were comparable. Finally, splitting the age group of 6–12 years into 6–10 (pre or early puberty) vs. 11–12 (pubertal) yielded similar results, with the exception that estimates were of slightly higher magnitude in the older children.

## 4 Discussion

Within a large paediatric primary care population, we found that both sleep duration and timing are associated with BMI‐defined overweight and obesity, though the relevance of duration versus timing depends on age. Specifically, short sleep duration was the most important predictor (i.e., largest effect size and highest precision) of paediatric overweight/obesity within the age range of 2–5 years, whereas later sleep midpoint and bedtime were more important predictors of overweight/obesity within the age range of 13–18 years. In the mid‐childhood range from 6 to 12 years, both short sleep duration and later sleep timing were associated with overweight/obesity.

Overall, our results point to independent roles of sleep duration and sleep timing on weight regulation in paediatric populations, which is consistent with some, although not all, prior literature [4, 5, 18]. There are plausible distinct mechanistic pathways. For example, both short sleep duration and delayed sleep timing could affect appetite and dietary quality, but in nuanced ways [19, 20, 21]. Short sleep duration could increase total energy intake and preference for highly palatable foods [22], while late sleep timing could be specifically related to a pattern of late‐night eating and breakfast skipping [23, 24], both of which are related to overall poorer diet quality. Similarly, both short sleep duration and later sleep timing could alter metabolism, but for different reasons. As one example, insufficient sleep leads to an overall reduction in deep sleep, the stage of sleep that growth hormone is released. Growth hormone plays a key role in metabolic function and is therefore a proposed link between insufficient sleep and higher adiposity [25]. Specific to sleep timing, a later bedtime is related to a higher likelihood of eating during the biological night, which can result in decreased energy expenditure and higher postprandial glucose levels [2, 26, 27]. This is one example of circadian misalignment, or a mismatch between the underlying circadian rhythms and external sleep/wake behaviours.

Our findings also extend the current knowledge base by illuminating potential differences in paediatric sleep and obesity associations according to age. Most prior studies focused on a smaller age range, not allowing for the age group comparisons that we made in the present study. We found that the quantity of sleep rather than the timing was more important at younger ages, which may reflect the overall higher sleep need, especially in toddlerhood [10]. In contrast, sleep timing was more predictive of obesity than sleep duration at older ages. A potential non‐causal explanation for a lack of association with sleep duration could be related to the measurement of sleep. In the estimation of sleep duration (simple calculation based on bedtime and wake time), we cannot account for wake after sleep onset (WASO); thus, for a teenager with a large WASO time, their sleep duration could be severely overestimated in our study. This misclassification bias may be more likely to affect the oldest age groups when difficulties with falling asleep and staying asleep (i.e., insomnia) can increase [28, 29]. In contrast, the estimation of sleep timing is not as affected by WASO. Moreover, one potential causal explanation for a distinct role of sleep timing during the teenage years may have to do with the fact that the pubertal transition causes changes in the circadian system, increasing their preference for eveningness [30]. External factors such as energy drink consumption and nighttime social media use, which may be unique to teenagers, can further delay bedtimes and/or exacerbate the circadian misalignment [28]. Interestingly, a recent study of 53 adolescents aged 14 to 18 years that used a gold‐standard measure of circadian misalignment (utilising dim light melatonin onset measurements) found that later DLMO and circadian misalignment, but not sleep duration, were related to higher BMI and energy intake, respectively [27].

Our results have potential clinical utility. Specifically, our data suggest that asking about the duration of sleep during well‐child visits may be more relevant at younger ages, whereas for older children and adolescents, specifically asking about bedtimes may be more pertinent. Framing the conversation specifically around bedtime may be relevant from a developmental perspective as well, since the adolescent stage coincides with more independence over choosing bedtimes and establishing their own bedtime routines [31]. Furthermore, although we recognise the superiority of sleep data collected via wearable devices in terms of accuracy [32, 33], self‐ or caregiver‐reported sleep duration and timing remain a cost‐effective and feasible option for widespread clinical use during well‐child visits [34].

This study leveraged a large electronic health record paediatric database spanning a wide age range that included repeated measures of sleep and BMI. Despite these key strengths, some limitations also need to be considered. As alluded to above, self‐reported or parent‐reported sleep often shows poor agreement with actigraphy‐assessed sleep, and differently according to child characteristics (e.g., age, race/ethnicity) [33, 35, 36]. Given the brief nature of the HSQ, only two questions on sleep were asked instead of the full validated CSHQ questionnaire. Thus, we did not have information on naps (most relevant to the youngest age group), the ability to distinguish sleep duration and timing on weekday and weekend nights, nor the presence of other sleep difficulties, including obstructive sleep apnea. We also did not have information on physical activity, which could act as a confounder, although we had information on some related behaviours including screen time and sugar‐sweetened beverage intake. The lack of a full food frequency questionnaire or 24‐h dietary recall precluded us from estimating usual daily energy intake, although this variable may serve as a mediator in the relationship between sleep and BMI rather than a true confounder. Pubertal status is another potential unmeasured confounder in the older age groups, since Tanner staging was not in our EHR database. Moreover, we acknowledge the limitation of using BMI as the sole marker of adiposity [37]. Another important contextual factor affecting generalisability is that the study period included the early stages of the pandemic (when schooling was done remotely), resulting in a wide distribution of both bedtimes and wake times. While this could potentially affect the generalisability of results, the greater variability of sleep times potentially enabled us to see a stronger association with sleep timing than can be observed when wake times are more fixed. Moreover, the reported sleep times may be more similar—and thus generalisable—to typical summer schedules. Other generalisability issues to note are that this study was limited to the US and therefore may not generalise to other settings. Similarly, there are unavoidable selection biases inherent in using clinical databases instead of sampling randomly from the target population. Finally, the possibility of reverse causation bias cannot be ruled out since sleep and BMI were measured at single points in time (albeit at multiple timepoints, depending on the patient). Future work that examines trajectories of sleep duration and timing across childhood in relation to concurrent changes in BMI would help to further elucidate these pathways.

In summary, results from this large paediatric well‐child database revealed that both short sleep duration and later sleep timing, that is, later bedtimes and sleep midpoints, were associated with higher BMI, but that these associations depended on age. Taken together, results suggest the need for additional research and clinical emphasis during well‐child visits on the importance of sleep timing in addition to sleep duration for weight status.

## Conflicts of Interest

The authors declare no conflicts of interest.

## Supporting information


**Data S1:** Supporting Information.

## Data Availability

The data that support the findings of this study are available on request from the corresponding author. The data are not publicly available due to privacy or ethical restrictions.
