# Supplementary material for: Role of Sleep Duration and Timing on Paediatric BMI Across Childhood and Adolescence: Do Both Matter?
Source: Pediatr Obes. 2025 Oct 21;21(1):e70064. doi: 10.1111/ijpo.70064 (PMC12579822; doi:10.1111/ijpo.70064)
Supplement: Supplementary file 1 — Data S1: Supporting Information. [file IJPO-21-e70064-s001.pdf]

**Supplementary Material**

**Title: Role of sleep duration and timing on pediatric BMI across childhood and adolescence: do both matter?**

**Authors:** Yundan Zhang[1], Joyce M. Lee[2], Karen E. Peterson[3,4], Jonathan A. Mitchell[5,6], Erica C. Jansen[3]

**Affiliations:**

[1]Department of Applied Health Science, School of Public Health, Indiana University

[2] Division of Pediatric Endocrinology, Susan B. Meister Child Health Evaluation and Research Center, University of Michigan

[3]Department of Nutritional Sciences, School of Public Health, University of Michigan

[4]Department of Environmental Health Sciences, School of Public Health, University of Michigan

[5] Division of Gastroenterology, Hepatology and Nutrition, Children's Hospital of Philadelphia

[6]Department of Pediatrics, University of Pennsylvania

**Corresponding Author:** Erica C. Jansen, PhD, MPH. Full address: 3863 SPH I, 1415 Washington Heights, Ann Arbor, Michigan, 48109. Email: janerica@umich.edu.

**Supplemental Figure 1.** Schematic of data cleaning process for sleep variables

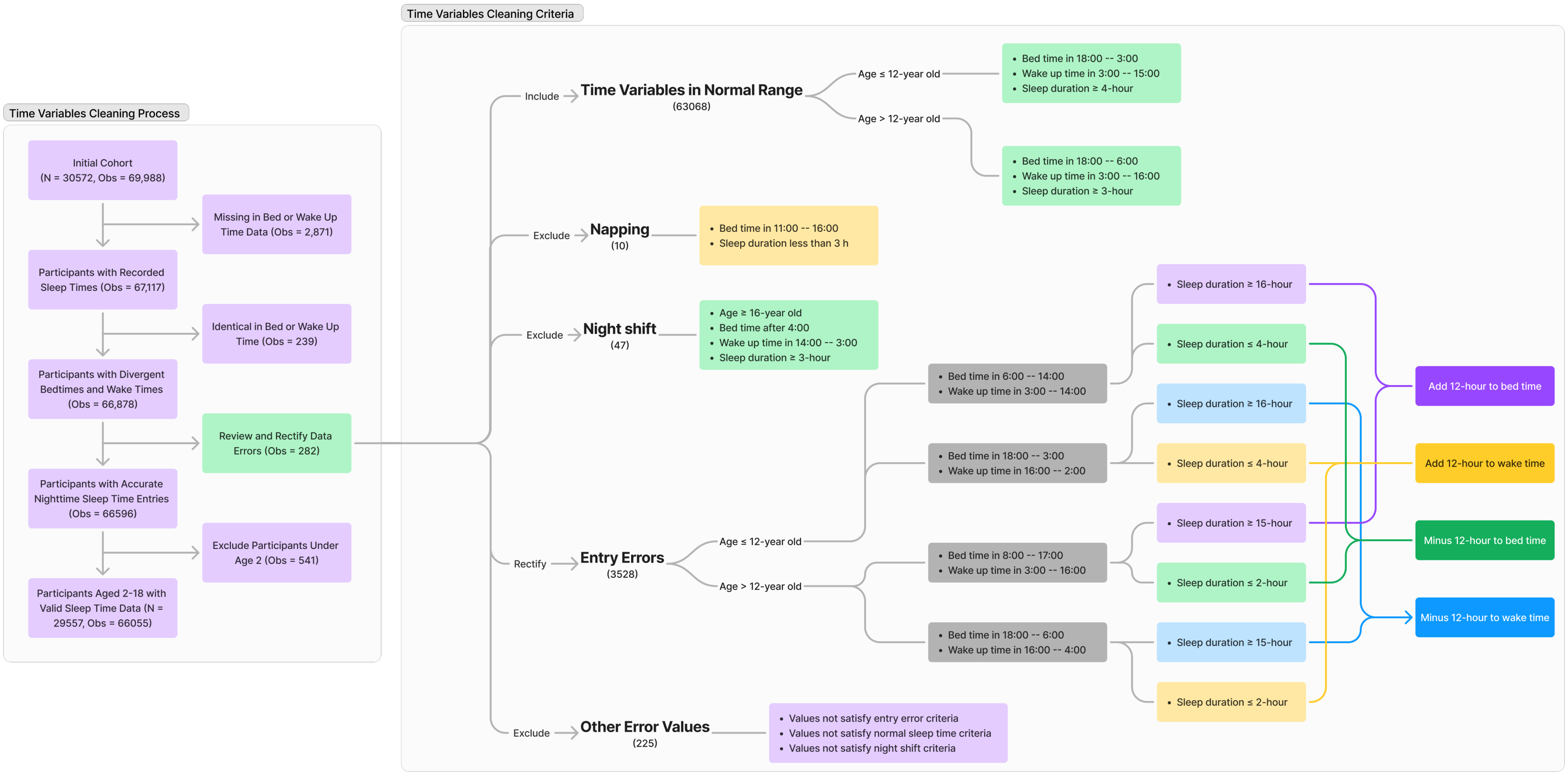

**Supplemental Figure 2.** Visualization of sleep timing and duration across age-based sleep midpoint quartiles

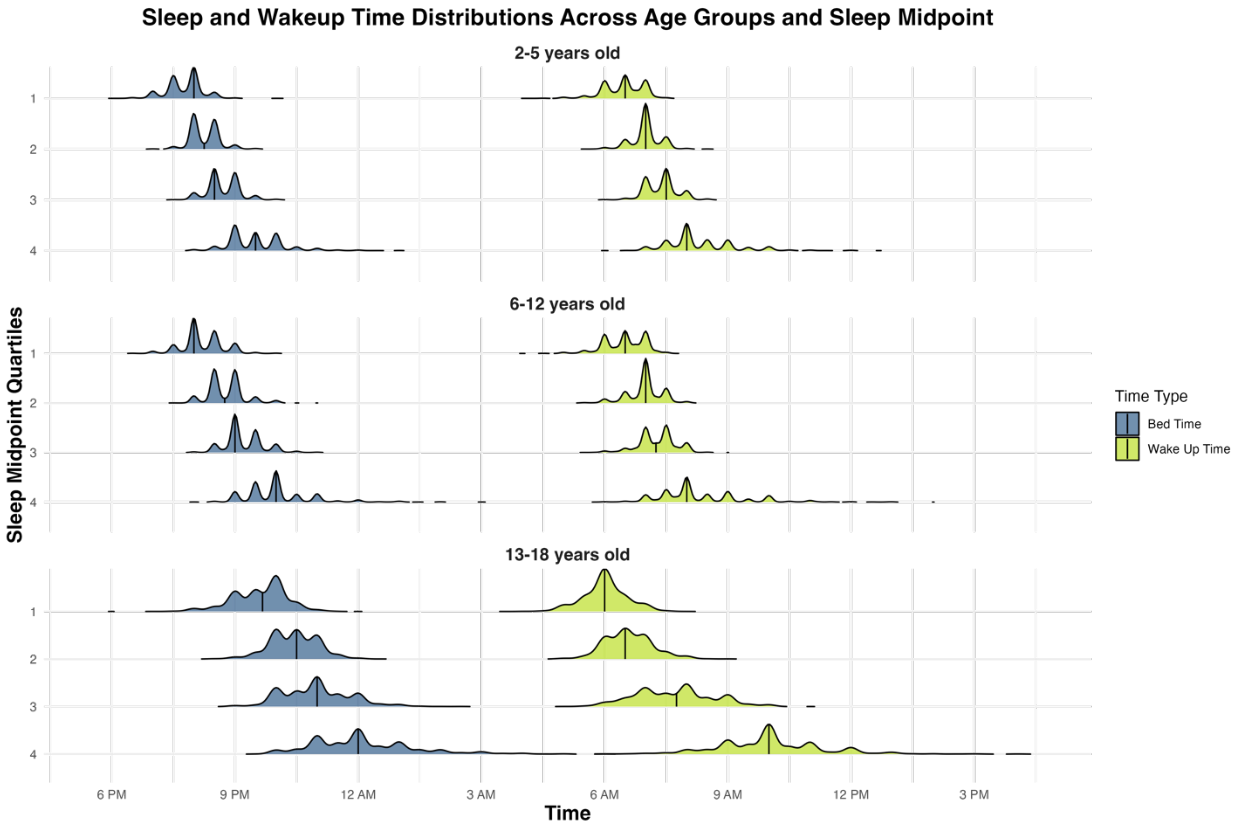


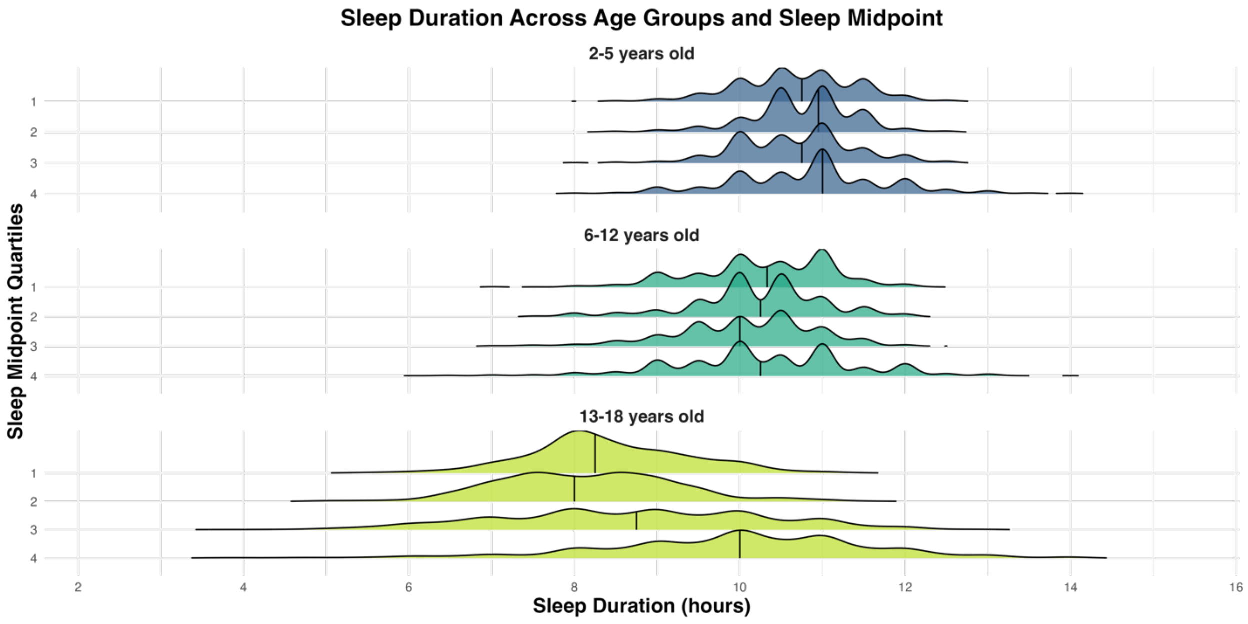

**Supplemental Figure 3.** Prevalence of overweight/obesity according to ages

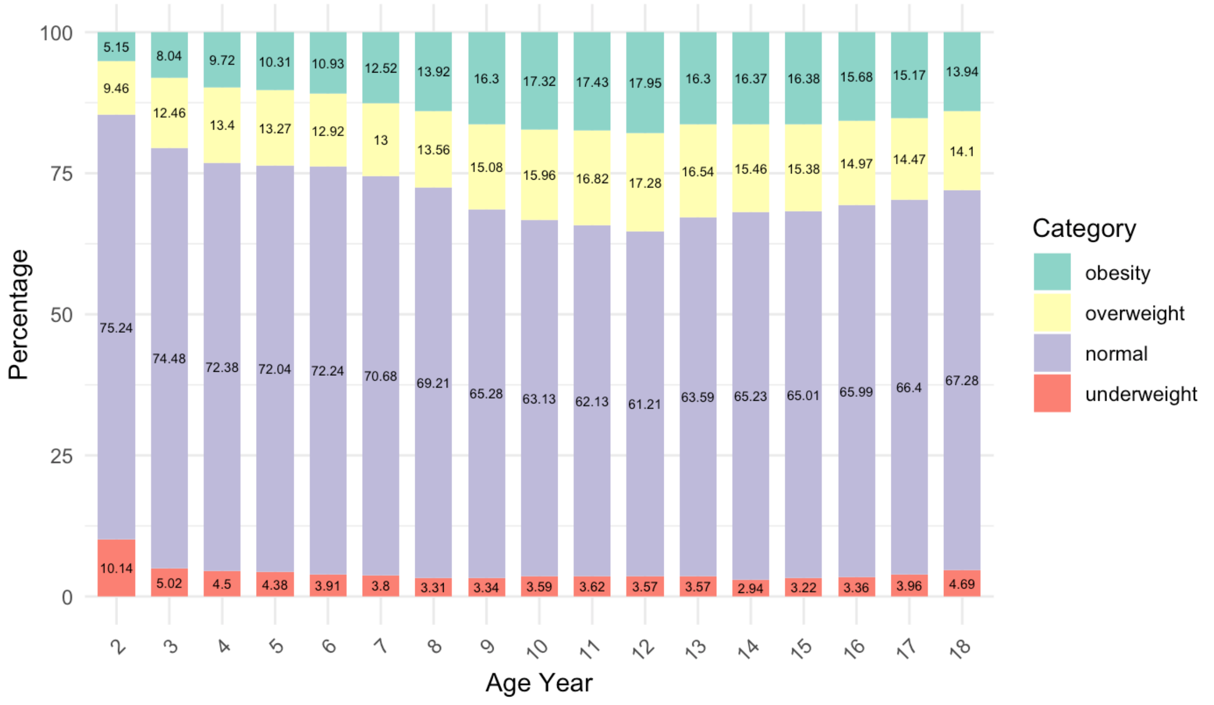

**Supplemental Table 1.** Number of exclusions under each error category[1]

| Types of Error | Count |
|---|---|
| Missing[1] | 4 |
| Exclude-Carried-Forward | 919 |
| Exclude-SD-Cutoff | 2 |
| Exclude-EWMA-Extreme | 12 |
| Exclude-EWMA-Extreme-Pair | 5 |
| Exclude-EWMA-8 | 174 |
| Exclude-EWMA-9 | 95 |
| Exclude-EWMA-11 | 19 |
| Exclude-EWMA-12 | 4 |
| Exclude-Min-Height-Change | 183 |
| Exclude-Max-Height-Change | 5 |
| Exclude-Pair-Delta-17 | 5 |
| Exclude-Pair-Delta-18 | 13 |
| Exclude-Single-Outlier | 36 |
| Exclude-Too-Many-Errors | 11 |
| Exclude-Too-Many-Errors-Other-Parameter | 3 |

[1] The total number of exclusions (1,490) does not exactly correspond to the number of exclusions shown in Figure 1 (1,473) because these exclusions were made separately for height and weight whereas Figure 1 shows exclusions based on BMI (i.e., both height and weight)

[2]The missing data are attributed to the absence of entries in the sex variable.

Definition:

1. Exponentially Weighted Moving Average (EWMA) calculation description.
   a. $\Delta Age_j = agedays_j - agedays_i$
   b. $EWMASD = SD_i = [\Sigma_{j \to n}(SD_j * ((5+|agedays_i - agedays_j|)^{\wedge}-1.5))] / [\Sigma_{j \to n}((5+|agedays_i - agedays_j|)^{\wedge}-1.5)]$
   c. For each of the three EWMASDs, calculate the dewma_*=SD-EWMASD
2. Carried forward. For the purposes of this analysis, any value that is identical to the preceding value for the same parameter and subject is considered carried forward. Because of variations in measurement, the chances of having identical measurements, even at an age/interval when little or no growth would be expected, is small.
3. Extreme errors with SD cutoffs. For this, a cutoff of |SD|>25 is used. Because of differences in SD and z score, there are some very extreme values with a |z|>25 that are implausible with an |SD|<25, so both are used to exclude extreme errors. This works better than using a lower value for the limit for |SD|.
4. Extreme errors with EWMA. Erroneous measurements can distort the EWMA for measurements around them. Therefore, if the EWMA method identifies more than one value for a subject and parameter that meets criteria for exclusion, we will only exclude the value that deviates the most from expected in any given step. Then we will repeat the entire process until no new measurements are identified that meet criteria for exclusion.
5. Moderate errors based on EWMA (including Exclude-EWMA-8, 9, 11, 12). This step is similar to step 3, with repeated exclusions of 1 value at a time, but with different criteria than step 3.

There are several criteria used as checks to make sure that values with a large dewma_* are not truly likely to be representative.

6. Absolute differences (including Min/Max-Height-Change). Once pairs of measurements with implausible amounts of absolute difference between them are identified, the EWMA will be used to determine which value is less likely to be representative and should be excluded. The EWMA analysis is adjusted depending on the number of available measurements: for pairs from datasets with three or more total measurements, the EWMA considers each measurement independently by excluding the counterpart from the calculation; for those with only two measurements, the decision is based on the absolute standard deviation of the measurements. The assessment utilizes growth velocity standards from the Tanner and World Health Organization (WHO) height velocity references to set benchmarks for plausible growth rates. The Tanner reference, which applies from ages 2 to 18 years, is used for annual growth velocity assessments. Conversely, the WHO standards offer guidelines for various shorter measurement intervals. When deciding between using Tanner and WHO references for a given pair, the choice is based on the age gap between measurements: WHO standards are preferred if the difference is less than nine months, otherwise, Tanner standards are used.
7. Exclusion for 1 or 2 measurements (including error for paired/single value). For pairs of values, several metrics are calculated: the absolute difference in the standard deviation (absd_tbc*sd), the absolute difference in age days (absd_agedays_*), and comparisons against the other parameter's standard deviation on the same age day (tbcOsd) or its median (median_tbcOsd). Depending on these calculated differences and the intervals between measurement days, different exclusion criteria (exc_* set to 17 or 18) are applied. Specifically, if the age day difference is greater than one year and the standard deviation difference exceeds three, or if the age day difference is less than one year and the standard deviation difference exceeds two, the measurement with the greater discrepancy from the median or the other parameter's value on the same day is excluded. For single values where exc_* is zero, the exclusion flag (exc_*) is set to 19 if the absolute standard deviation exceeds certain thresholds relative to either tbcOsd or median_tbcOsd, depending on their availability.
